# Exploiting protein flexibility to predict the location of allosteric sites

**DOI:** 10.1186/1471-2105-13-273

**Published:** 2012-10-25

**Authors:** Alejandro Panjkovich, Xavier Daura

**Affiliations:** 1, Institute of Biotechnology and Biomedicine (IBB)Universitat Autònoma de Barcelona (UAB), 08193 Cerdanyola del Vallès, Spain; 2, Catalan Institution for Research and Advanced Studies (ICREA), 08010 Barcelona, Spain

## Abstract

**Background:**

Allostery is one of the most powerful and common ways of regulation of protein activity. However, for most allosteric proteins identified to date the mechanistic details of allosteric modulation are not yet well understood. Uncovering common mechanistic patterns underlying allostery would allow not only a better academic understanding of the phenomena, but it would also streamline the design of novel therapeutic solutions. This relatively unexplored therapeutic potential and the putative advantages of allosteric drugs over classical active-site inhibitors fuel the attention allosteric-drug research is receiving at present. A first step to harness the regulatory potential and versatility of allosteric sites, in the context of drug-discovery and design, would be to detect or predict their presence and location. In this article, we describe a simple computational approach, based on the effect allosteric ligands exert on protein flexibility upon binding, to predict the existence and position of allosteric sites on a given protein structure.

**Results:**

By querying the literature and a recently available database of allosteric sites, we gathered 213 allosteric proteins with structural information that we further filtered into a non-redundant set of 91 proteins. We performed normal-mode analysis and observed significant changes in protein flexibility upon allosteric-ligand binding in 70% of the cases. These results agree with the current view that allosteric mechanisms are in many cases governed by changes in protein dynamics caused by ligand binding. Furthermore, we implemented an approach that achieves 65% positive predictive value in identifying allosteric sites within the set of predicted cavities of a protein (stricter parameters set, 0.22 sensitivity), by combining the current analysis on dynamics with previous results on structural conservation of allosteric sites. We also analyzed four biological examples in detail, revealing that this simple coarse-grained methodology is able to capture the effects triggered by allosteric ligands already described in the literature.

**Conclusions:**

We introduce a simple computational approach to predict the presence and position of allosteric sites in a protein based on the analysis of changes in protein normal modes upon the binding of a coarse-grained ligand at predicted cavities. Its performance has been demonstrated using a newly curated non-redundant set of 91 proteins with reported allosteric properties. The software developed in this work is available upon request from the authors.

## Background

Proteins can be regarded as the functional building blocks of life, carrying out and coordinating almost all biological processes. Tight regulation of these processes is fundamental in all kingdoms of life and allostery represents one of the most commmon and powerful means of modulating protein activity [1]. Allostery can be defined as the regulation of a protein’s function by binding of an effector molecule at a site which is not the active site. Its relevance was emphasized decades ago by Jacques Monod, when he referred to allosteric regulation as the ‘second secret of life’, second only to the genetic code [2]. Even though allostery and its often intrincate nature have captured the interest of researchers since the initial discoveries more than half a century ago (for a review see [3]), most allosteric mechanisms are still not completely understood [1]. At present, allosteric phenomena are being intensively studied for their potential as target mechanisms for the development of new classes of therapeutics [4].

Expanding drug-design through allostery opens up an unexplored territory of novel potential therapeutic solutions, beyond what has been already covered by the classic, active-site oriented drug-development approach. An important factor fueling interest in allosteric drugs consists in their characteristic advantages compared to traditional active-site inhibitors. For example, allosteric sites tend to be under lower sequence-conservation pressure than active sites, facilitating the design of highly specific drugs and reducing the risks of toxicity or side-effects [5-7]. To explain this briefly, if the pathogen’s active site is very well conserved in nature it may share important structural features with the human homologue, which could be then bound and inhibited as well by the antimicrobial drug causing toxic side-effects on the patient. Thus, lower levels of evolutionary conservation at ligand-binding sites may allow for more selective drugs. Furthermore, allosteric drugs may not only inhibit but also increase target-protein activity, enabling novel therapeutic possibilites as seen for example in the activation of glucokinase by allosteric drugs, a potential treatment for type 2 diabetes mellitus [8,9]. On the same line, traditional drugs may be complemented by allosteric effectors, as observed in the case of aminoglycoside phosphotransferase where a previously unknown binding site could be exploited to allosterically counteract antibiotic resistance [10].

However, the field of allosteric-drug design is rather young and the amount of allosteric drugs known today is still marginal [7]. For example, at the time of this writing a query in DrugBank [11] for the term ‘allosteric’ returns 7 results, while ‘inhibition’ returns 483 entries. This may be in part due to the intrinsic difficulties in understanding allosteric mechanisms and to the lack of systematic studies on the topic [12]. Only recently the first initiative to store and organize information on allosteric cases has surfaced in the form of the AlloSteric Database (ASD) [13]. By browsing ASD it becomes apparent that part of the difficulty in studying allosteric systems lies in the large degree of variety found among them, as there are many ways in which protein activity can be affected allosterically [12,14]. A textbook example is the one provided by glucose-induced glucokinase, in which the ligand triggers a conformational change allowing the active site to become functional [15]. In other cases the presence of the allosteric ligand triggers the formation of the biologically active protein complex (*e.g.* GTP cyclohydrolase stimulatory complex [16]). A protein illustrating the variety and complexity allosteric mechanisms may reach is ribonucleotide reductase. This protein presents two different allosteric sites: one affects the enzyme catalytic rate and the other alters its specificity allowing the enzyme to switch substrates [17]. Furthermore, allosteric signals may also propagate solely by altering protein dynamics, without a detectable conformational change [18,19].

In the context of such diversity, unveiling common patterns beneath allosteric phenomena could increase their potential for therapeutic exploitation, stimulating the design of allosteric drugs. We postulate that the first step in such a procedure would be to computationally detect or predict the presence and location of protein allosteric sites, to allow further focusing of drug-screening processes on selected protein targets down the pipeline. The algorithm should be able to pinpoint which proteins are sensible to allosteric regulation. However, if as already suggested any dynamic protein has the potential to be regulated allosterically [20], then the method should indicate the location of putative allosteric sites on the protein. Based solely on sequence, it would be very hard to predict the location of allosteric sites as it has been done by homology on active sites [21,22], because the evolutionary pressure for sequence conservation on allosteric sites is generally much lower and harder to detect, if at all present [3,23].

Until now, much of the research in the field has been focusing on the conformational changes induced by allosteric signals. The group of Jeffrey Gray studied conformational changes upon allosteric activation [24] and expanded this research by analysing the networks of quaternary and tertiary motions on which allosteric communication relies [25]. Following a similar line, a very interesting and thorough study was published where different parameters were interrogated in terms of their potential to indicate which protein residues are involved in transmitting the allosteric signal, on the basis of experimental mutation data [26]. The results from these analyses aim at defining the particular pathway of residues that mediate the allosteric communication. However, other authors have argued that this may not be the case *in vivo*, where multiple effector sites may be present on the protein acting through multiple signaling pathways [27]. In general, recent studies aggree in the idea that allostery is mainly a thermodynamic process and among the different protein properties that are involved in allosteric phenomena, flexibility (*i.e.* protein dynamics) stands out as the most significant one [3,28-31].

Following this line of thought, Ming and Wall developed a theoretical framework to study allosteric effects by comparing the dynamics of bound and unbound protein-ligand pairs [32]. They further refined their methodology and tested its ability to predict functional ligand-binding sites (not necessarily linked to allosterism) on a set of 305 protein-ligand complexes of known structure [33]. Very recently, two other approaches partially aiming at predicting allosteric sites have been published by Mitternacht and coworkers. In a first article they describe a geometric measure that helps at locating biologically functional ligand-binding sites, while a second one describes a more elaborate measure called ‘binding leverage’, related to protein dynamics, which appears useful at locating biologically relevant binding sites including allosteric sites. They tested this last feature on 15 allosteric proteins [34,35], observing different results for specific proteins and concluding that regulatory sites may be identified without previous experimental knowledge on conformational changes. However, these studies were not completely focused on allosteric sites and did not benefit from the larger data set now available at ASD [13].

Even though the previously cited articles represent an important step forward in the understanding of allostery, we consider that further research is needed if allosteric sites are to be predicted with the same coverage and precision as active sites [21,22]. The first thing we did in this direction was to integrate more than one hundred allosteric entries available at ASD. Among the multiple allosteric mechanisms known and the different effectors (other proteins, small-molecules, phosphorylation, etc), we chose to focus on small-molecule ligands, as these are the best candidates to be mimicked by therapeutic drugs [4,14]. Moreover, the approach presented here is based on the idea that changes in protein flexibility upon ligand binding can be related to allosteric and regulatory effects [1,24,36-38]. A simple computational way to estimate protein structural flexibility is the use of Normal Mode Analysis (NMA) [32,35,38,39]. In this case, however, we were not interested in measuring absolute flexibility values but the change in flexibility that occurs when a ligand binds to the protein structure in a particular location, in a similar fashion to the approach developed by Ming and Wall [32]. Once we had gathered and filtered allosteric proteins of known structure, we tested if changes in flexibility could be linked to the presence of the allosteric ligand. Experiments were performed using different molecular representations of the small-molecule ligands, and across different ranges of normal modes. Moreover, as a control we simulated the presence of ligands in alternative binding sites. This helped in parameterizing the methodology and made it applicable to cases where there is no *a priori* knowledge on the allostery the protein may present. Besides evaluating the overall results on a set of allosteric proteins, we took a closer look into particularly interesting cases.

## Results and discussion

### Gathering structural data on allosteric sites

To study allosteric sites from a structural perspective we first gathered the available data. We started by integrating the 146 allosteric site entries that were, at the time of this writing, annotated in the AlloSteric Database (ASD) [13] with another 72 allostery examples we had previously found in the literature. We proceeded to filter and cluster the data set as described in the Methods section to avoid overrepresentation [40] and low quality structures, turning the inital 213 cases into a total of 91 representative proteins where both the structure and location of the allosteric ligand are known.

### Allosteric-ligand presence affects protein flexibility

Our first experiment aimed at quantifying the number of proteins in our data set that undergo a significant change in flexibility when the allosteric ligand is bound. However, known allostery cases show large diversity in their mechanisms [12] and we did not expect a positive result on the complete data set, since in many cases the allosteric effect may not be primarily driven by changes in local or overall flexibility but specific conformational changes, oligomerization or other mechanisms may be more relevant [41].

As explained in the Background section, we have chosen to estimate flexibility using Normal Mode Analysis (NMA). When applying NMA, calculated low-frequency modes reflect large collective oscillations of the protein structure and high-frequency modes reflect small local fluctuations [39]. Even though for most cases it has been shown that low-frequency normal modes are better descriptors of allosteric effects [42], we made no *a priori* assumption on the set of normal modes that would be more appropriate to detect an allosteric effect upon ligand binding for the ample protein set studied here. Thus, we decided to explore this parameter by using different ranges of normal modes, as described in the Methods section.

We used the calculated normal modes to predict B-factors [39], as this is a standard quantity for the estimation of protein flexibility [38]. Briefly, NMA calculations were performed for proteins in our data set both in the presence and absence of the allosteric ligand. For each protein, C*α* B-factors derived from both conditions were compared and considered to be significantly different if the Wilcoxon-Mann-Whitney test returned a *p-value* < 0.05.

**Figure 1:**
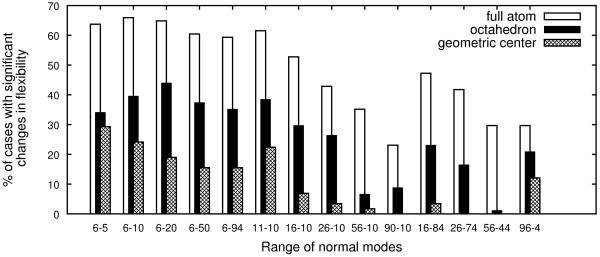
**Ligand simulation.** Identification of changes in flexibility upon ligand binding when using different ligand representations. Normal-mode range X-Y corresponds to the initial X modes being skipped and the next Y modes being taken into account.

The results are displayed in Figure 1 and show that for the majority of the data set protein flexibility is significantly affected by the presence of the allosteric ligand. For most cases the effect was only observed when low-frequency normal modes were considered, as expected [35]. However, there are exceptions like the ribonucleotide reductase from *Thermotoga maritima* ([PDB:1XJF]), for which the allosteric effect has been described to be related to the local stabilization of three loops in the structure [4,17] and was captured only by high-frequency normal modes in our calculations.

### Effect of ligand representation on the NMA results

We performed a second experiment to measure how different the results from this approach would be if we used a simplified molecular representation instead of the full-atom ligand, given the fact that knowledge on the ligand structure may not always be available. Moreover, a predictive approach that does not require information on the ligand molecule has a much larger field of application (*e.g.* structural genomics) paving the way for the discovery of novel and pharmacologically interesting allosteric sites. Another interesting possibility that would open up is the detection of serendipitous allosteric sites, which despite having no natural ligand effectively become an allosteric site given the presence of an appropriate ‘opportunistic’ ligand [1].

We tested two representations of ligands: a single dummy atom located at the ligand geometric center and a set of 6 dummy atoms located at the vertices of an octahedron around the geometric center, as explained in the Methods section.

Figure 1 shows that for most cases the single dummy atom at the ligand geometric center is not able to trigger a significant change in flexibility during the simulations, while the octahedron exerts an effect much closer to that of the full-atom ligand. From a methodological point of view, simulating ligands in a simplified form allowed us to perform control experiments which are described below.

### Predicting ligand-binding pockets and selection of normal mode range

To further develop a predictive approach, we used the LIGSITE^*cs*^program [43] to predict the putative ligand-binding sites on the protein structure. Different programs are available for this task with very good performance in general as shown in recent reviews [44-46]. We chose LIGSITE^*cs*^because pockets are predicted based only on the shape of the protein surface; programs incorporating more parameters (*e.g.* evolutionary conservation, druggability) could have improved but also biased our results.

We predicted the location of up to 8 ligand-binding pockets per protein and performed NMA to check if any of the predicted pockets had a significant effect upon protein flexibility when occupied by a small-molecule ligand, as described in the Methods section.

**Figure 2:**
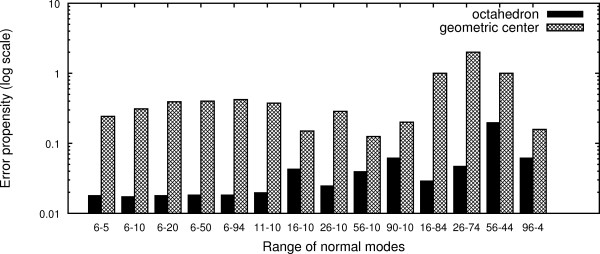
**Error propensity estimation.** Ratios between the number of cases with 1 or 2 significant pockets and the number of cases with 7 or 8 significant pockets. Normal-mode range X-Y corresponds to the initial X modes being skipped and the next Y modes being taken into account.

A pocket that presents no ligand (*i.e.* appears empty in the original structure) may nevertheless display a significant change in overall flexibility if occupied by a ligand representation when performing the normal-modes calculation. It would be wrong to consider this directly an error, since native ligands may exist that bind this pocket even if there is none present in the particular experimental structure under study. A few examples are mentioned in the next section, where we found pockets that affect protein flexibility and, although they are indeed not allosteric sites, they are active sites or other biologically relevant sites. Nevertheless, to guide the definition of the model we needed an error propensity estimate for the different parameters tested, i.e. range of normal modes and ligand molecular representation. If all pockets predicted on the protein surface would be found to affect significantly the flexibility, the corresponding parameters would be rendering the method too sensitive (low specificity) and prone to present false positives. Based on this argument, we estimated error propensity (*ep*) for each range of normal modes and the two ligand representations using the following *ad hoc* equation:

(1) $$\mathrm{ep}=\frac{p_{7}+p_{8}+1}{p_{1}+p_{2}+1}$$

where *p*_*x*_ is the number of cases in which *x* pockets were predicted to be significantly affecting the overall protein flexibility. Note that this equation does not formally stand for an error but simply gives an idea of the likelihood of having false positives. The results are displayed in Figure 2 and show that the octahedron representation, combined with the lowest frequency normal modes, leads to a higher specificity (lower number of pockets significantly affecting overall dynamics) than the single dummy atom at the geometric center. We then decided to continue our work using the octahedron representation of ligands together with normal modes in the range 6-20.

### Overall performance when predicting allosteric sites

At the time of this writing, no large-scale study attempting the prediction of the allosteric-site location in known allosteric proteins has been published. Recent work by Demerdash and coworkers aimed at predicting the residues involved in the propagation of allosteric signals within a protein structure for a set of 16 different proteins [26]. Quite distinctly, our method follows a drug-discovery oriented approach where the intention is to pinpoint specific protein pockets that present a high potential for affecting biological function. From that perspective our method is comparable to the one developed by Ming and Wall for finding functional sites, as it exploits NMA to assess the differences in flexibility between ligand-bound and unbound states of a protein [33,47]. However, their approach differs from ours in multiple major points, including the sampling of protein sites, the parametrization of probes and of their interaction with the protein and the approach by which the perturbation of protein dynamics is assessed. The method described in this paper is also similar to the approach recently published by Mitternacht and coworkers, where they measured the ‘binding leverage’, or ability of a binding site to couple to the intrinsic motions of a protein, on a set of 15 allosteric proteins [35].

In this context, any pocket with a biological regulatory role would be suited for the analysis, but we chose to focus on allosteric sites since these are possibly the most interesting, albeit complex, regulatory sites to approach. Starting from a data set containing 91 proteins, we measured the rate of success of our approach to identify allosteric sites as follows. First, we discarded a total of 33 proteins for which no single LIGSITE^*cs*^predicted pocket matched the allosteric site, leaving a total of 58 cases to work with (63,7%). The rational for discarding these proteins is that the present analysis is not concerned with the ability of a specific program to detect a cavity but with the ability of our approach to identify the cavity, among those detected, that corresponds to the allosteric site. Indeed, it has been previously observed that not all allosteric sites are predicted to be potential ligand-binding cavities by common algorithms [35]. There can be different reasons for this, for example the allosteric site may be deeply buried in the protein, may display a planar shape or be located at the interface of subunits, making it difficult for the pocket-prediction algorithm to detect its presence.

A total of 464 pockets were predicted on the surface of the 58 proteins (8 per protein). The chance of randomly selecting an allosteric site is low, given that only 13% of these pockets (one per protein) matched the location of an allosteric site (*i.e.* its center less than 5 Å away from the allosteric ligand; if more than one pocket matched the ligand position within this cut-off, the closest was chosen). After performing the analysis of normal modes, 117 pockets display a significant effect on the overall protein flexilibity upon ligand binding (set F in Table 1). The chance of success (positive predictive value) more than doubles with the incorporation of this analysis, with 27% of these 117 pockets matching an allosteric site. Furthermore, we integrated these results with our previous work on protein-pocket conservation by selecting pockets that display at least 50% structural conservation, as defined previously [23]. Interestingly, considering protein conservation alone (set S in Table 1) results in a slightly lower positive predictive value than considering only flexibility. While the two measures show the same specificity, using the effect on flexibility as criterion leads to a slightly higher sensitivity than using the structural-conservation feature. The double-filtered set, combining the effect on flexibility with a high structural conservation (set FS), contains only 36 pockets, of which 15 (42%) match an allosteric site (Table 1). This represents a nearly four times larger positive predictive value than ‘random’ selection within the 464 identified cavities, at the expense of reducing further the sensitivity of the approach, i.e. decreasing drastically the number of false positives but increasing also the number of false negatives.

**Table 1:** Prediction results on protein allosteric sites

Set	TP+FP	TP	FP	FN	Sensitivity	Specificity	Accuracy	PPV
Total	464	58	406	0	1.00	0.00	0.13	0.13
F	117	32	85	26	0.55	0.79	0.76	0.27
S	108	24	84	34	0.41	0.79	0.75	0.22
FS	36	15	21	43	0.26	0.95	0.86	0.42
c123	174	44	130	14	0.76	0.68	0.69	0.25
c123F	74	29	45	29	0.50	0.89	0.84	0.39
c123S	55	22	33	36	0.38	0.92	0.85	0.40
c123FS	30	14	16	44	0.24	0.96	0.87	0.47
c1	58	26	32	32	0.45	0.92	0.86	0.45
c1F	42	22	20	36	0.38	0.95	0.88	0.52
c1S	25	15	10	43	0.26	0.98	0.89	0.60
c1FS	20	13	7	45	0.22	0.98	0.89	0.65

Results on this table refer to the subset of 58 proteins for which LIGSITE^***cs***^was able to predict a ligand-binding pocket in the position of the allosteric site. TP: true positive; TN: true negative; FP: false positive; FN: false negative; PPV: positive predictive value. Sensitivity: TP/(TP+FN); specificity: TN/(TN+FP); accuracy: (TP+TN)/(TP+FN+TN+FP); PPV or precision: TP/(TP+FP). The total number of pockets considered, predicted by LIGSITE^***cs***^, is 464 (8 per protein). F corresponds to sets including a change in flexibility as selection criterion; S corresponds to sets including high structural conservation as selection criterion; c123 refers to sets considering only the three largest pockets predicted by LIGSITE^*cs*^; c1 refers to sets considering only the largest predicted pocket.

However, it will not be common to select up to eight pockets per protein as potential allosteric sites. A researcher working on a particular protein without *a priori* knowledge on its regulatory mechanism will probably keep the first three largest pockets predicted by default [43] or, as Thornton and coworkers explain for the case of active sites [21], the largest pocket will usually be the best bet. In those two scenarios, the tendency shown for the complete set of predicted pockets is conserved (Table 1). When keeping the first 3 pockets (set c123), the chance to match an allosteric site (positive predictive value) goes from 25% to 39% when using the flexibility criterion and up to 47% when incorporating structural conservation as well. Out of the 58 allosteric sites, however, 14 are not found within the c123 set. Likewise, when selecting only the first and largest pocket, the inital success rate goes from 45% to 52% when considering the effect on flexibility upon binding (set c1F) and to 65% when structural conservation is also required. Note that between sets FS and c1FS the number of false positives decreases by three-fold, while only two additional false negatives are added.

We considered only allosteric sites as desirable matches. However, other pockets with biological functions were matched by our criteria, as described further below on a few particular examples. The performance of this approach might be improvable using other pocket prediction programs or a combination of them. However, performance of pocket prediction methods does not vary largely, as shown by a recent large-scale comparison [45]. Our study represents the largest test to date (58 non-redundant proteins in complex with their corresponding small-molecule allosteric ligands) proving the concept that changes in overall flexibility upon ligand binding are relevant identifiers for some allosteric sites, and these effects can be captured in many cases with the simple approach described here. In addition, we further show (see also [23]) that evaluation of the structural conservation of the candidate pockets may contribute as much to the identification of the allosteric site.

### Biological examples

As mentioned in the Background section, allostery can work through many different mechanisms. Thus, we consider it important, besides the overall results presented above, to explain the results for a few proteins in more detail. The following section should help to better illustrate the relevance of incorporating a flexibility measure when studying allosteric systems and predicting the location of allosteric sites.

#### Glyceraldehyde 3-phosphate dehydrogenase

Aldehyde dehydrogenases (ALDH) are found across all kingdoms of life. They play a vital role in multiple cellular processes, including glycolysis, detoxification and embryogenic development. A distinct family within the ALDH superfamily consists of the non-phosphorylating glyceraldehyde-3-phosphate dehydrogenases (GAPN), which catalyze the phosphate-independent irreversible oxidation of glyceraldehyde 3-phosphate (GAP) to 3-phosphoglycerate using NAD(P) as a cosubstrate. Unlike other proteins in the GAPN family, the enzyme of the hyperthermophilic Archaeum *Thermoproteus tenax* (Tt-GAPN) is regulated by a set of inhibitors (NADH, NADP(H) and ATP) and activators (AMP, ADP, glucose 1-phosphate and fructose 6-phosphate (F6P)) which decrease or increase, respectively, the affinity for NAD. This suggests that Tt-GAPN plays a crucial role in regulating the carbohydrate catabolism in *T. tenax*[48].

All different activators bind to the same allosteric site, which is located more than 20 Å away from both the active site and the cosubstrate-binding site of any monomer of the tetramer [49]. The activator binding site is located at the interface between the tetramerization domain and the cosubstrate binding/catalytic domains. It is also observed that the allosteric ligands are in direct contact with 3 or even four monomers in the protein complex, indicating a role in the stabilization of the complex. This role probably combines with the detected effect on flexibility to influence enzyme kinetics, as no large conformational change is observed when comparing the ligand-bound and ligand-free structures besides a rearrangement of the tetramerization domain with respect to the cosubstrate binding/catalytic domain [49].

**Figure 3:**
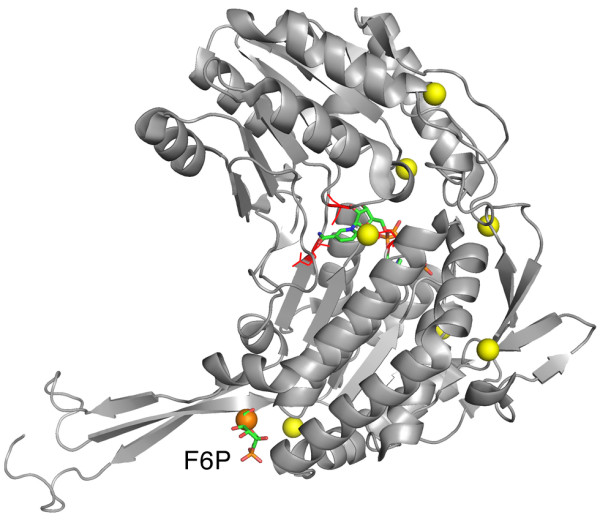
**Glyceraldehyde 3-phosphate dehydrogenase.** Predicted pockets and ligands on *Thermoproteus tenax* glyceraldehyde 3-phosphate dehydrogenase (TtGAPN). Only a single protein monomer is shown ([PDB:1UXR]). NADPH at the active site and activator fructose-6-phosphate (F6P) at the allosteric site are shown in ‘sticks’ representation, while residues in red correspond to the CSA [21] active site annotations. Predicted pockets (geometric centers) are shown in ‘spheres’ representation, the pocket in orange color affected protein flexibility significantly according to our simulations, while yellow did not.

In our analysis, PKT5 (the fifth largest pocket predicted) matched the location of the allosteric effector F6P, as shown in Figure 3. When a ligand was simulated occupying this pocket, using the octahedron representation, the overall flexibility of the protein was significantly affected on all ranges including the lowest frequency modes (*p-value*<=0.001). No other pocket presented the same behaviour (Figure 3), not even the largest pocket (PKT1), which matches the position of cofactor NADP. Given the ‘hinge-like’ position of the activator binding site and the variety of ligands that it can accomodate, we consider this case a good example to speculate that the actual position of the ligand in the structure plays a major role in its effect on the protein activity, beyond the particular chemical properties of the ligand itself that may be important for binding.

#### PDK1 kinase

**Figure 4:**
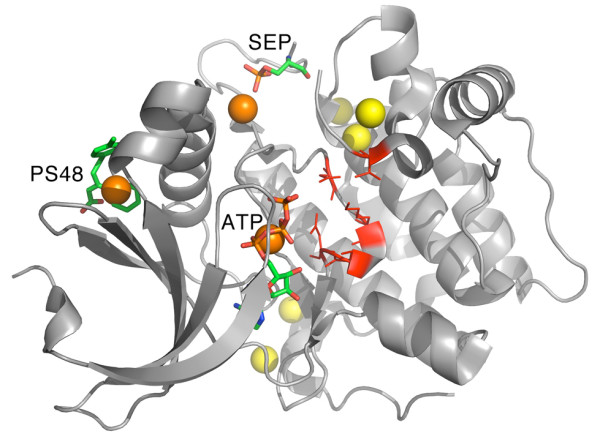
**PDK1 kinase.** Predicted pockets and ligands on PDK1 kinase ([PDB:3HRF]). Ligands (ATP, allosteric effector PS48) and the modified residue phosphoserine (SEP) are shown in ‘sticks’ representation, residues in red correspond to the CSA [21] active site annotations. Predicted pockets (geometric centers) are shown in ‘spheres’ representation, orange pockets affected protein flexibility significantly according to our simulations, but yellow did not.

PDK1 kinase is a key regulator of AGC kinases, which play crucial roles in physiological processes relevant to metabolism, growth, proliferation and survival [50]. This protein is regulated allosterically by the binding of a phosphopeptide which Biondi and coworkers managed to mimic with a low-molecular-weight activator [51] and further solved the structure of the complex [52]. As shown in Figure 4, the largest pocket (PKT1) matches the binding site of ATP. In our analysis, PKT1 affected protein flexibility significantly on most normal-mode ranges. The second largest pocket predicted (PKT2) matches the location of the allosteric activator (PS48) at the HM/PIF binding site. According to the analysis, based on the lowest-frequency normal modes (6-5 range) PKT2 significantly affects overall protein flexibility if occupied by a ligand.

Another predicted pocket (PKT7), appears to significantly affect protein flexibility on most normal-mode ranges when occupied during the NMA. This pocket is not occupied by any ligand in the original structure ([PDB:3HRF]), but it does match the position of a phosphoserine (SEP) in the activation loop of PDK1, as shown in Figure 4. Also in this pocket, residue THR226 is considered a crucial element of the allosteric mechanism of this protein, given that mutation of this residue inhibits activation without inhibiting binding [52]. These results indicate that stabilization of this protein region would have an effect on the overall flexibility of the protein, linking it to a regulatory function which correlates with what has been observed previously based on deuterium exchange and other experimental procedures [52]. The other 5 pockets predicted on this structure were not found to significantly affect protein flexibility.

#### HIV reverse transcriptase

Non-nucleoside reverse transcriptase inhibitors (NNRTIs) are key elements of the so-called HAART (Highly Active Antiretroviral Therapy) multi-drug treatments against HIV-1 infection. However, rapid mutation of HIV-1 compromises the efficacy and durability of HAART. This high mutation rate fuels the need to discover novel agents with better activity profiles against HIV-1 reverse transcriptase (RT) and its most common mutants. In this context, Anthony and coworkers have developed substituted tetrahydroquinolines which are potent allosteric inhibitors of HIV-1 RT and some of its key mutants [53].

**Figure 5:**
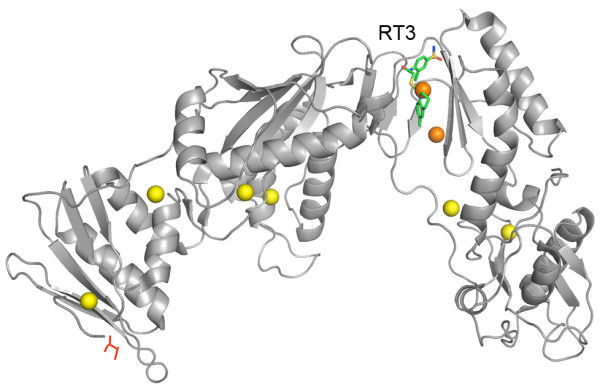
**HIV reverse transcriptase.** Predicted pockets and ligands on HIV reverse transcriptase ([PDB:3I0R]). Compound RT3 at the allosteric site is shown in ‘sticks’ representation, while residues in red correspond to the CSA [21] active site annotations. Predicted pockets (geometric centers) are shown in ‘spheres’ representation, the pocket in orange color affected protein flexibility significantly according to our simulations, while yellow did not.

In our normal-mode analysis the two significant pockets matched the position of the allosteric ligands, as displayed in Figure 5. The allosteric site is located in a ‘hinge-like’ position between domains, a position which has even been exploited for the engineering of regulatory sites as well [54]. A ligand bound in this position would easily perturb the low frequency modes of vibration of the protein, thus affecting its overall flexibility and subsequently altering protein function. All other pockets predicted on this structure were found not to affect protein flexibility significantly, meaning that a hypothetical blind drug-design approach focused on the significant pockets from the NMA would have been successful. This is an excellent example showing that the combination of pocket prediction and NMA may pinpoint the location of the allosteric/regulatory site based solely on structural data.

#### L-lactate dehydrogenase

When glycolysis takes place under anaerobic conditions, pyruvate is reduced to L-lactate, a reaction that is catalyzed by L-lactate dehydrogenase (LDH). In contrast to their mammalian counterparts, some bacterial LDHs display allosteric regulation by fructose 1,6-bisphosphate (FBP) [55]. Iwata and co-workers solved the structure of *Bifidobacterium longum* LDH in both active (R) and inactive (T) states, co-crystallized with the allosteric activator [56]. A significant difference can be observed between the B-factors of both structures, suggesting an overall change in flexibility being part of the allosteric mechanism.

**Figure 6:**
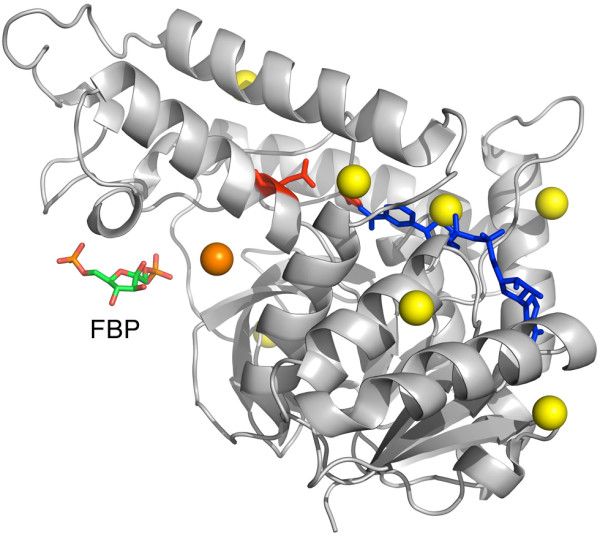
**L-lactate dehydrogenase.** Predicted pockets and ligands on L-lactate dehydrogenase ([PDB:1LTH]). Fructose-1,6-bisphosphate (FBP) at the allosteric site is shown in ‘sticks’ representation as well as NAD which is colored blue, while residues in red correspond to the CSA [21] active site annotations. Predicted pockets (geometric centers) are shown in ‘spheres’ representation, the pocket in orange color affected protein flexibility significantly according to our simulations, while yellow did not.

We mentioned this protein in our previous work [23], where we found the allosteric site to be structurally conserved although no signal of sequence conservation was found. In the current analysis, the only pocket that perturbed the overall flexibility of LDH when we simulated the presence of a ligand was the second largest pocket (PKT2), which is also the one closest to the allosteric site, as displayed in Figure 6. We did not consider this case as a ‘match’ in the large-scale results shown in Table 1 because the pocket geometric center is 6.6 Å away from the allosteric ligand, thus failing the pre-defined threshold of 5 Å. However, after visual inspection we considered this case relevant because the ligand is occupying the same large pocket, even if it is not located precisely at the pocket center defined by LIGSITE^*cs*^.

No other pocket on this protein displayed an effect related to flexibility according to our calculations when considering the normal mode range 6-20, not even those pockets matching the location of the active site or other ligands.

Given that animal LDHs are not regulated allosterically, this protein/pocket could be an excellent target for antimicrobial compounds. To further explore this idea, we analyzed the human LDH homolog ([PDB:1I10]) as well, which shows a sequence identity of 37.7% and a local RMSD of 1.04 Å according to the SUPERPOSE web server [57] when compared to *Bifidobacterium longum* LDH. On the human protein, which is not regulated allosterically, the pocket equivalent to the allosteric site in the bacterial homolog did not produce a significant effect on flexibility according to our calculations. It is remarkable that a coarse approximation such as this (based on C*α*s and NMA) is able to distinguish that the presence of the allosteric ligand has a significant effect on the bacterial protein flexibility but not on its human homolog.

## Conclusions

In this article we have proposed a very simple approach exploiting changes in protein flexibility upon ligand binding to predict the presence and location of allosteric sites. We tested the methodology on a non-redundant set of 58 proteins achieving in the best case a success rate (positive predictive value) of 65%, with a sensitivity of 0.22. Furthermore, we analyzed four cases in more detail, revealing how the coarse-grained approach described here is able to capture the effect triggered by the allosteric ligand, matching the current literature. The structural analysis proposed here could help medicinal chemists and other researchers on their way through the promising field of allosteric-drug design.

## Methods

To ensure that the quality and nature of the selected structural data was appropriate to our study, we discarded structures with a resolution lower than 3 Å or with a G-Factor lower than -1, as calculated by PROCHECK [58]. We conservatively defined a non-redundant data set to avoid possible bias in the results that may arise from overrepresentation of any protein family [40]. Clustering was performed with the BLASTCLUST program [59] using a threshold of 30% sequence identity, which grouped the 213 initial entries into 91 groups. We then selected the highest resolution structure of each group as its representative and defined a non-redundant data set which contained a total of 91 distinct allosteric proteins, for which the structure and location of both the allosteric site and ligand were known.

Normal mode analysis (NMA) was performed on the protein crystallographic structures with and without a probe ligand, in its three different representations (full atom, octahedron and geometric center, see below). The simplified ligand representations (octahedron and geometric center) were alternatively placed in each of the eight predicted pockets. The NMA was based on the implementation of Sanejouand and coworkers [39,60] using the programs PDBMAT and DIAGRTB. The calculation involves the diagonalization of the mass-weighted Hessian (**H**) of the potential energy function *V *. Following Tirion’s Elastic Network Model [61], the potential energy *V * is simply described as a set of harmonic springs of equal strength *k* linking every pair of C*α* atoms with a distance smaller than *R*_*c*_in the crystallographic structure:

(2) $$V=\underset{\overset{r_{\text{ij}}^{0}<R_{c}}{i<j}}{\sum }k{\left(r_{\text{ij}}-r_{\text{ij}}^{0}\right)}^{2}$$

where $r_{\mathrm{ij}}^{0}$ is the Euclidean distance between atoms *i* and *j* in the crystallographic structure and *R*_*c*_ and *k* were given in this study the values 10 Å and 1 Kcal mol^−1^Å^−2^, respectively. Note that this energy function was designed in such a way that it does not require energy minimization of the X-ray structure prior to the normal-mode calculation since the X-ray structure is the minimum of the function. Although this method uses very gross approximations (reduction to C*α* atoms, extremely simple energy function, no solvent), it has proven to perform surprisingly well in front of both more complex approximations and experimental data (B-factors) [39,60,62].

The eigenvectors and eigenvalues of **H** correspond to the normal modes, characterizing the direction and amplitude of the vibrational motion, and frequencies of vibration, respectively. They can be used to calculate mean-square displacements of the atomic cartesian coordinates *x* (〈*x*^2^〉) as:

(3) $$\langle x_{i}^{2}\rangle =\frac{K_{B}T}{m_{i}}\overset{n_{v}}{\underset{j=1}{\sum }}\frac{a_{\text{ij}}^{2}}{w_{j}^{2}}$$

where *x*_*i*_ is the coordinate *i*, *m*_*i*_ the corresponding mass, *K*_*B*_ Boltzmann’s constant, *T* the temperature, *n*_*v*_ the number of modes considered, *w*_*j*_/2*Π* the frequency of normal mode *j* and *a*_*ij*_the coordinate *i* of normal mode *j*. The resulting mean-square radial displacements of atom positions 〈*r*^2^〉 can in turn be used to estimate atomic B-factors as:

(4) $$B=\frac{8\Pi^{2}}{3}\langle r^{2}\rangle$$

Low-frequency modes reflect large collective or delocalized motions in the protein structure, while high-frequency modes reflect small vibrations in localized regions. We estimated B-factors for the ligand-bound and unbound protein structures using different ranges of normal modes to explore this variable. Ranges were named using two numbers X-Y: Starting from the low frequency modes, X is the number of modes that are skipped and Y is the number of normal modes that are taken into account. The first six normal modes, with zero frequency, are of no interest as they represent rigid-body translation and rotation. The ranges tested were: 6-5, 6-10, 6-20, 6-50, 6-94, 11-10, 16-10, 26-10, 56-10, 90-10, 16-84, 26-74, 56-44 and 96-4.

We prepared protein coordinate files for NMA as follows: (1) Protein chains in direct contact with the allosteric ligand (*i.e.* multiple residues within 3.0 Å) were selected and atoms belonging to other chains or molecules in the structure were removed. (2) The LIGSITE^*cs*^program [43] was used to predict up to 8 pockets per structure. (3) After pocket prediction, protein structures were parsed to keep C*α*atoms only.

We took the first 100 normal modes for each protein and ligand representation: **apo**, the C*α* only ‘apo’ protein crystallographic structure (allosteric ligand is not present); **ligand**, same protein structure as in ‘apo’ but including the allosteric ligand (or a simplified molecular representation) in the allosteric site; **PKTX**, same protein structure as in ‘apo’ plus a simplified molecular representation of a ligand occupying the predicted pocket number X (1 to 8).

In the last case, the molecular representation of the ligand was located at the pocket geometric center, as predicted by LIGSITE^*cs*^.

The ligand molecule during NMA was simulated in different ways: **full atom**, all atoms in the ligand molecule are included in the calculation; **geometric center**, a single dummy atom located at the ligand-pocket geometric center is considered; **octahedron**, the ligand’s presence is simulated by a dummy atom positioned at the geometric center and six extra dummy atoms located at 4 Å distance from the center on both sides of each axis (*i.e.* forming the vertices of a regular octahedron).

For each protein-ligand pair, calculated B-factors for the C*α* protein atoms of the **apo** structure were compared to those obtained for the same atoms in the configurations including real or simulated ligands to test for significant changes in flexibility using the Wilcoxon-Mann-Whitney test. Differences with a *p-value*<0.05 were considered significant.

## Competing interests

The authors declare that they have no competing interests.

## Authors’ contributions

AP and XD conceived the study and wrote the manuscript. AP carried out the computational work. Both authors read and approved the final manuscript.
